# Current and Future Insights in Organic–Inorganic Hybrid Materials

**DOI:** 10.3390/polym16213043

**Published:** 2024-10-29

**Authors:** Jesús-María García-Martínez, Emilia P. Collar

**Affiliations:** Polymer Engineering Group (GIP), Polymer Science and Technology Institute (ICTP), Spanish National Research Council (CSIC), C/Juan de la Cierva, 3, 28006 Madrid, Spain

The text below outlines some current and future possibilities for organic–inorganic hybrid materials. This third installment builds on the success of the previous Special Issues and presents new original contributions and approaches to the topic [1,2,3,4]. These investigations align with the definition of hybrid materials recommended by the IUPAC (International Union of Pure and Applied Chemistry). This definition signifies that a hybrid material comprises a close mixture of inorganic, organic, or both components, typically interpenetrating scales of less than one micrometer [5]. The potential for organic–inorganic hybrid materials to enhance properties in various fields is vast, offering substantial research and design opportunities for material scientists. However, it is necessary to note that functional hybrid materials are not just physical mixtures [6,7,8,9,10]. They are nanocomposites at the molecular scale, with at least one organic or inorganic component, having a characteristic length on the nanometer scale (a few Å to several tens of nanometers) [7]. The properties of hybrid materials are not just the sum of the individual contributions of their components but also arise from the strong synergy created by a hybrid interface [8,9,10].

This synergy is a key aspect of hybrid materials, highlighting the unique properties that can be achieved through careful design. These, such as enhanced electrical, optical, mechanical, separation capacity, catalysis, sensing capability, and chemical and thermal stability properties, make hybrid materials a valuable area of research. The nature of the inorganic–organic interface, including the types of interactions present, the surface energy, and the presence of labile bonds, plays a strong role in controlling these properties. Additionally, it must be noted that hybridization is a multifaceted strategy. In some cases, conducting organic polymers just act as a solid polymeric support for active species. In contrast, in other hybrid systems, the activity of organic and inorganic species combines to reinforce or modify each other. However, in every case, the work on these hybrid materials involves the underlying use and sometimes even the explicit search for synergy. Hybrid organic–inorganic materials, in general, represent the natural interface between two worlds of chemistry, each with very significant contributions to the field of materials science and each with characteristic properties that result in distinct advantages and limitations. Researching the topic of hybrid materials has challenges and opportunities. The main challenge is synthesizing hybrid materials that keep or enhance each component’s best properties while reducing their limitations. Undertaking this challenge allows one to develop new materials with synergic behavior, improving performance and novel valuable properties. Indeed, hybrid materials frequently involve a combination of components thoroughly studied in their respective fields. Still, they provide an additional dimension to their properties when becoming part of the hybrid compound [11].

Various functional hybrid materials can be categorized into two main families depending on the nature of the interface, combining organic components and inorganic materials [7,8,10,11,12]. Class I deals with hybrid systems where the organic and inorganic parts interact by weak bonds, including Van der Waals, electrostatic, or hydrogen bonds. Class II indicates hybrid materials in which covalent or ionic-covalent chemical bonds link these components. Many hybrid materials combine jointly strong and weak interfaces [1,2,3,4,10,13,14]. Still, due to the significance of the presence of strong chemical bonds on the final properties, these hybrids are grouped into Class II. Hybrid Class I compounds have exciting features such as ease of material synthesis, easy removal of the organic phase to create functional architectures by self-assembly, et cetera. However, there is increasing development of Class II hybrid materials due to the advantages of covalent bonds between organic and mineral components, including the potential to synthesize entirely new materials, minimization of phase separation, and better definition of the organic–inorganic interface [7,8,9,10]. The effective grafting of organic functionality to the inorganic network also avoids a drawback of the hybrid compounds of Class I, which is the potential departure of organic components while the material is in use. Furthermore, categorizing organic–inorganic hybrid systems into Class-I and Class-II, based on the type of interactions between the phases, highlights these materials’ diverse and promising nature. Combining weak and strong interactions in the same hybrid system opens new avenues for innovation and development. In summary, organic–inorganic materials are shown as multi-component compounds with at least one component in the nano-metric size domain. This yields significantly enhanced properties, positioning them as valuable components in various applications. To finalize, in essence, it can be said that the organic–inorganic materials are multi-component compounds with at least one of their organic (the polymer) or inorganic components in the nano-metric-size domain, which confers the material as a whole of greatly enhanced properties respecting the constitutive parts in isolation [6,7,8,9,10].

It is well worth mentioning here the so-called hybrid fiber reinforced polymer composites (HFRPCs) since they are organic–inorganic hybrid advanced materials combining two or more different types of fibers (organic and inorganic) within a polymer matrix, aiming to leverage the strengths of each type of fiber, addressing their individual limitations to achieve enhanced mechanical properties and performance, making them appropriate for automotive, aerospace, and construction applications due to their outstanding performance and cost-effectiveness derived both from the combination of materials and the processing operations choice [15,16,17,18].

Of the nineteen manuscripts submitted to this Special Issue, just 13 were published after the rigorous revision processes of *Polymers*. This Special Issue, therefore, includes these highly relevant and exciting works that are of utmost importance in this scientific field. Each article compiled in this volume fully matches the topic’s fundamentals, underscoring their significance. Since this editorial aims not to elaborate on each text but to encourage the reader to browse them in depth, these contributions have been briefly described. Consequently, a brief resume of each one is reported to awaken interest in each of the contributions to this exciting Special Issue of *Polymers* rather than provide an exhaustive description of the articles themselves.

So, the contribution by Wang, Li, and coworkers [19] introduces a unique approach to designing and preparing high-performance polymer-based electrolytes for solid-state energy storage devices. The authors have developed a novel double-network PE based on the nonhydrolytic sol–gel reaction of tetraethyl orthosilicate and in situ polymerization of zwitterions. The resulting material, with its high strength and stretchability, represents a significant advancement in the field. This is largely due to the efficient dissipation of energy in the inorganic network. This unique property also allows it to act as a Lewis acid to adsorb trace impurities, resulting in an electrolyte with a high electrochemical window. The elastic characteristics of the polymer network further enhance its performance. Notably, the new PE demonstrates excellent interface compatibility with a Li metal electrode, an essential requirement for solid-state energy storage devices.

In their article, Solechan et al. [20] present a significant study on biocomposite scaffolds obtained from a blend of polylactic acid (PLA) and polycaprolactone (PCL) using cold isostatic pressing and incorporating hydroxyapatite (nHA) as an osteoconductive filler (0 up to 30%). This research, focusing on medical applications, aims to determine the effects of nHA on compound performance. Different characterization techniques were employed from both the micro and macro scales, such as FTIR, XRD, SEM, density, porosity, tensile, and flexural properties. The study concludes that incorporating nHA into the PLA/PCL blend induces an irregular structure, making the crystallization process more challenging. Higher amounts of nHA result in more porous materials. These findings have practical implications for the development of biomaterials in medical applications.

In their paper, Spiridonov et al. [21] presented synthesized nanocomposites of cerium-containing nanoparticles stabilized by carboxymethyl cellulose (CMC) macromolecules through a novel and elegant method. These products were further characterized, aiding in determining the type of crystal structure of inorganic nanoparticles and the mechanism of nanoparticle formation. They demonstrated and confirmed that neither the size nor the shape of the nanoparticles present in the nanocomposites depends on the ratio of the initial reagents, reassuring the method’s robustness.

The article by Peponi, López, et al. [22] is a meticulous study that delves into the design and development of multifunctional fibers. This involves the incorporation of functionalized nanoparticles into matrices obtained by spinning techniques. The authors present a green protocol for obtaining functionalized silver nanoparticles, using it as a reducing agent. These nanoparticles, once obtained, were integrated into PLA solutions to explore the production of multifunctional polymeric fibers by centrifugal force-spinning. The study focused on nanoparticle concentration between 0 and 3.5 wt%, investigating the effect of nanoparticle incorporation and fiber preparation method on morphology, thermomechanical properties, bio-disintegration, and antimicrobial behavior. The authors’ conclusion that 1% of nanoparticles is the optimal amount for enhancing thermomechanical behavior while conferring high antibacterial activity to the PLA fibers and that 2% significantly enhances the material’s shape memory underscores the meticulousness of this study.

In this article authored by Mahato, Abaimov, and colleagues [23], they have presented a comprehensive overview of the application of nanofiber polymeric veils as toughening interleaves in fiber-reinforced composite laminates. They note that the interest of these veils is to prevent delamination caused by the poor out-of-plane properties of composite laminates. They also identify and discuss the toughening mechanisms induced by polymeric veils. The results and conclusions claimed to be helpful in all the stages of the material designs, from the material selection to the modelization of the delamination process, provide a solid foundation for further research and application.

The article by Leonés, López, Peponi, and coworkers [24] is dedicated to the obtention of filaments based on polylactic acid (PLA) doped with varying amounts of magnesium microparticles. The authors employed a two-step extrusion process to investigate the impact of processing on the thermal degradation of the filaments. They also studied the in vitro degradation of the filaments and their subsequent 3D printing. The study culminated in the successful production of micro-composites via a double-extrusion process, with no degradation of the materials and a commendable dispersion of the microparticles into the PLA matrix, all without any chemical or physical modification of the microparticles.

Thus, the contribution by Maqableh et al. [25] has developed some analytical methods to evaluate the bond strength of fiber–matrix systems. For this purpose, they investigate the debonding mechanism of a fiber–silicone pull-out specimen and further validate the experimental data using 3D-FEM and a cohesive element approach. The comparison between the experimental values and the results from the finite element simulations shows that the proposed cohesive zone model accurately reproduces the experimental results, being considered almost identical to the experimental observations about the interface. This accuracy of the cohesive zone model reassures the reliability of the research.

The article authored by Godelmoula and colleagues [26] is a significant study on using carbon fiber-reinforced polymers (CFRPs) in fabricating complex geometries through selective laser sintering. It is crucial to note that while carbon fiber reinforcement enhances the mechanical properties of polymers, it also reduces tribological wear resistance, necessitating fillers as solid lubricants. The authors’ exploration of graphite-filled carbon fiber-reinforced polyamide 12 (CFR-PA12) specimens, prepared using the selective laser sintering process, provides a deep understanding of the composite’s dry sliding friction and wear characteristics, leading to engaging concluding remarks.

The aim of the investigation of the paper by Aranha, Carvalho et al. [27] is the determination of water sorption in hybrid polyester/glass fabric/jute fabric composites molded via compression and vacuum-assisted resin transfer molding, obtained at different stacking sequences and water sorption testing at different temperatures. The authors concluded that the manufacturing process does not affect water sorption at the saturation point, as the main factors influencing the absorbing moisture are the presence and content of jute fibers in the system, which are jointly affected by the immersion temperature. Additionally, in contribution by Aranha, Rivera, and colleagues [28], these authors have studied the mechanical behavior under tensile stress of neat and hydrothermally aged samples at different temperatures, evaluating fracture surfaces by SE, and it was concluded that exposure to the aqueous ambient led to a reduction in mechanical properties, both for the molding technique and the stacking sequence, observing a broad spectrum of defects such as delamination, fiber pull-out, fiber/matrix detachment, voids, and matrix removal correlated to the experimental conditions followed.

The article authored by Tellez et al. [29] has investigated encapsulated caffeine (CAF), which has anti-cellulite properties, in zirconium-based metal–organic frameworks (MOFs) by liquid-assisted milling, resulting in different textural properties and chemical functionalization. These capsules have been incorporated into recycled polyamide 6 (PA6) and a biopolymer based on polylactic acid (PLA) using extrusion. The resulting materials have been fully characterized, confirming the caffeine encapsulation, the preservation of caffeine during the extrusion process, and the good contact between the polymer and the MOFs. This research opens up exciting possibilities for using these materials in various applications, inspiring further exploration and development in the field.

The research performed by Cerrada et al. [30] meticulously investigated composites based on poly(3-hydroxybutyrate) (PHB) and mesoporous SBA-15 silica, a study conducted with utmost care and precision. The thermal stability, phase transitions, and crystalline details of these composites were studied in great detail, focusing on the confinement of the PHB polymeric chains in the silica’s mesopores. The influence of nano-silica in the composites’ thermal, morphologic, and dynamic mechanical performance not only confirms the observed confinement but also provides a comprehensive understanding of the influence of the filler in temperatures above the glass transition.

Finally, the last article, authored by Peponi, López, and coworkers [31], is devoted to a comprehensive study that thoroughly explores the bioactivity and antibacterial behavior of PLA-based electrospun fibers doped with both MgO and Mg(OH)_2_ nanoparticles (NPs). The study meticulously tracks the evolution of these fibers in terms of morphology, infrared spectra, X-ray diffraction, and visual appearance. The authors discuss the bioactivity of hydroxyapatite growth after 28 days of immersion in simulated body fluid (SBF), noting an increase in the number of precipitated crystals with the amount of both NPs. The chemical composition of the precipitated crystals, characterized in terms of the Ca/P molar ratio after T28 of immersion in SBF, indicates the presence of hydroxyapatite on the surface of the reinforced fibers, along with a decrease in the average diameter of the PLA-based fibers. The study also reveals the promising antibacterial activity of the MgO and Mg(OH)_2_ nanoparticles in the fibers against Escherichia coli and Staphylococcus aureus, providing a comprehensive understanding of their behavior.

To conclude, and as the guest editors of this captivating Special Issue, we are excited to announce that “Organic–Inorganic Hybrid Materials” has emerged as a crucial framework in the field of polymer science and technology, both currently and in the near future. This excitement fuels our anticipation for the fourth Special Issue on the topic, slated for 2025 in POLYMERS. We are now open for submissions and eagerly await contributions from those who share our passion for this vibrant scientific field.
